# Glances at the Hospitals

**Published:** 1902-09-13

**Authors:** 


### GLANCES AT THE HOSPITALS.

### DERBYSHIRE ROYAL INFIRMARY.

The total income amounted to £8,783, and the expenditure to £9,917, showing an adverse balance of about £1,134. This sum was carried to the extraordinary expenditure, and in addition to it £5,470 was spent on additions and repairs. The annual subscriptions have increased from £3,231 to £3,651, and there is also some increase in the Saturday and Sunday Funds. The cost per patient per week was £1 1s. 6d.; and the cost per patient was £3 16s. 10d. There were 150 in-patients at the beginning of the year, and 2,101 were admitted, making a total under treatment of 2,251. Of these 1,388 were discharged cured, 34 were made out-patients, 393 were discharged relieved, 98 for various reasons, and 180 died; 158 remained in the hospital at the end of the year. The medical and surgical statistics are very well arranged. There were 75 cases of typhoid fever, of whom 61 recovered and 14 died. In 4 of the latter bronchitis existed, in 4 pneumonia, in 3 thrombosis, in 1 perforation, and in 1 meningitis. Fourteen cases of pneumonia were under treatment with the result that 12 were cured, 1 relieved, and 1 died. The operation for perforated gastric ulcer was performed in 4 cases, 2 being cured and 2 died. Ovariotomy was performed 11 times, in all cases successfully. There were 49 cases of cataract, of which 36 were cured, 10 relieved and 3 not relieved. A complete list of operations is given with the results. The total number performed was 783. Of these 685 were successful. In 53 the patients were relieved, in 10 not relieved, and 35 died. The anæsthetic table shows that chloroform was administered 433 times, ether 585 times, a mixture of chloroform and ether 92 times, nitrous oxide 42 times, and cocaine 117 times.

### FORSTER GREEN HOSPITAL FOR CONSUMPTION, BELFAST.

Owing to the increasing demands for admission the revenue was insufficient last year, and the account shows a deficiency on the year's working of £419. In addition to this there was a deficiency in the previous year of £214, so that there was a total deficiency of £633. To meet this the President, Mr. Forster Green, has given £250, and a special effort, which we hope will be successful, is being made to raise the balance, so that the small endowment fund need not be encroached upon. On January 1st the hospital contained 23 patients. During the year 142 cases were admitted, making a total of 165 under treatment. Of the cases admitted 15 per cent. were slightly affected, 35 per cent. were moderately affected, and 45 per cent. were very seriously affected. The results of the open-air treatment of the above show that 42 per cent. were much improved, 26 per cent. improved, and in 18 per cent. there was no improvement. In phthisis the conditions which more especially bear upon the results of treatment are the age of the patient and the extent of the disease. The first cannot be altered by any hospital arrangements, but had the Belfast Hospital been twice as large, and twice as rich, many of those returned as very seriously affected might have been treated in the first stage of the disease, when chances of recovery are immeasurably greater than in the third. It is surely not too much to expect that the population of a rich and populous city like Belfast will come forward and subscribe handsomely to this truly Christian work. We venture to suggest that next year's report should contain a short tabulated statement of the inclusive cost per patient per week, and the average stay of each patient in the Sanatorium.

### QUEEN'S HOSPITAL, BIRMINGHAM.

The income amounted to £9,606, and the expenditure to £11,268. The number of in-patients was 2,169, and the out-patients 28,895. The cost of each in-patient amounted to £3 10s. 7d., and the average residence in the hospital was 21 days. The daily average number of patients was 121, and the death-rate was 1·4 per cent. The statistical tables show the sex and age of the patients and the results of treatment. For instance 20 men and 9 women suffered from enteric fever, 23 of these were cured and 6 remained under treatment. Among the surgical diseases we notice that the operation for appendicitis was performed 23 times by one of the surgeons, 21 of these being successful. In another surgeon's wards the same operation was performed 28 times without a death, and in a third it was performed 14 times with 1 death. Cæsarian section was performed successfully.

### BRADFORD ROYAL INFIRMARY.

The income reached the sum of £10,146, but the expenditure was more than that, and the governors had to apply a legacy of £500 to the current account. In spite of that there was an adverse balance of £1,685, and the governors may well say that the finances form the subject of most serious consideration. Such difficulties may be met in a variety of ways. The subscriptions may be increased, the number of patients may be reduced, or perhaps economy exercised in some directions. On looking over the subscription lists we noticed that something under £400 is subscribed by the friendly societies and by the workpeople themselves. This source of revenue ought to be at least doubled. The duration of each patient's stay in the hospital has been reduced from an average of 27 days to one of 23½ days. This is a step in the right direction. The cost of food, maintenance, etc., has, however, gone up from £17 13s. in 1898 to £19 12s. in 1901, and in the same period the cost per day per patient has increased from 11½d. to 12¾d. The cost per bed occupied has fallen from £62 12s. to £60 14s. The daily average number of in-patients was 160, and the death-rate was 7·3 per cent. Excluding those who died within 24 hours of admission the percentage falls to 5·28. The governors mention some things which the hospital needs, and which they think citizens might be glad to supply. Among these are lifts for the two wings, equipment of the operating theatre, and a Röntgen-ray apparatus. There is a nursing school in the hospital, and during the year 147 applications were received from would-be nurses. Of these 29 were taken on trial, and 15 proceeded to the full three years' course. The medical and surgical tables are well compiled.

